# GenoEye: A machine learning‐based framework for the prediction of intermediate eye color phenotypes

**DOI:** 10.1111/1556-4029.70403

**Published:** 2026-07-02

**Authors:** Davide Dalfovo, Giorgia Pallafacchina, Gianluca Occhi, Alberto Marino, Giovanni Vazza, Christian Faccinetto, Alessandro Romanel

**Affiliations:** ^1^ Department of Cellular, Computational and Integrative Biology (CIBIO) University of Trento Trento Italy; ^2^ Neuroscience Institute Italian National Research Council CNR Padova Italy; ^3^ Department of Biomedical Sciences University of Padova Padova Italy; ^4^ Department of Biology University of Padova Padova Italy; ^5^ Reparto Carabinieri Investigazioni Scientifiche di Parma, Sezione Biologia Parma Italy; ^6^ Present address: OncoRay–National Center for Radiation Research in Oncology, Faculty of Medicine and University Hospital Carl Gustav Carus Technische Universität Dresden, Helmholtz‐Zentrum Dresden‐Rossendorf Dresden Germany

**Keywords:** eye color prediction, forensic DNA phenotyping, intermediate eye color, machine learning, SNPs

## Abstract

Forensic DNA phenotyping (FDP) enables the prediction of externally visible characteristics from genetic data, providing valuable investigative leads. While current approaches achieve high accuracy for blue and brown eye color, the prediction of intermediate phenotypes such as green and hazel remains challenging, particularly in Southern European and Mediterranean populations. In this study, we developed GenoEye, an interpretable machine learning framework for three‐category eye color prediction based on an expanded SNP panel, from which a final 37‐SNP predictive signature was derived. The model was developed and evaluated on a cohort of 363 Italian individuals using a gradient boosting approach, with independent data used for performance assessment. GenoEye achieved high predictive performance for blue and brown eye color (AUC up to 0.97), while improving the classification of intermediate phenotypes (AUC = 0.79), a category where existing tools show limited sensitivity. Comparative analyses under multiple decision strategies demonstrated that GenoEye provides consistently higher sensitivity for intermediate eye color while maintaining high specificity. To support practical application, GenoEye is implemented as a web‐based tool with integrated SHAP‐based interpretability. These results indicate that GenoEye improves the resolution of intermediate eye color prediction and provides a robust and interpretable framework for forensic applications.

**Highlights**

- A novel 37‐SNP signature improves prediction of eye color.
- Machine learning improves sensitivity for challenging intermediate phenotypes.
- The model outperforms standard forensic tools in an Italian cohort.
- SHAP‐enhanced web app delivers transparent and interpretable forensic leads.

## 1 INTRODUCTION

Forensic DNA phenotyping (FDP) is a valuable methodology in forensic science, providing investigative leads when standard DNA profiling does not yield a match from a database. This approach allows for the prediction of an individual's externally visible characteristics (EVCs), generating information about the physical appearance of an unknown person [1]. Pigmentation traits, specifically eye, hair, and skin color, are particularly well‐suited for prediction due to their high heritability and well‐characterized genetic basis [2]. Research has extensively focused on eye color, and its variation is largely determined by a set of key genes, including *HERC2*, *OCA2*, *SLC24A4*, *SLC45A2*, and *IRF4* [2].

Following these genetic discoveries, several predictive models have been developed. The most widely used is the IrisPlex system, which employs a statistical model based on six SNPs to calculate probabilities for an individual having blue, brown, or intermediate eye color [3]. The success of this model led to the HIrisPlex and HIrisPlex‐S systems, which incorporate additional markers for the simultaneous prediction of hair and skin color [4]. Other methods, including the *Snipper* classifier and various decision‐tree models, also exist [5, 6]. All these tools demonstrate high accuracy, often exceeding 90% for the classification of distinct blue and brown eye colors in many populations.

However, a persistent challenge in FDP remains the accurate classification of *intermediate* eye colors, such as green and hazel. This difficulty stems from the complex polygenic architecture of these phenotypes, which result from the combinatorial effects of multiple loci beyond the primary markers used to distinguish blue and brown pigmentation [2, 7]. This challenge is especially pronounced in populations of Mediterranean and Southern European ancestry, where the prevalence of these complex shades is significantly higher than in Northern European groups. In these demographic contexts, which serve as a critical model for understanding complex pigmentation, allele frequencies at key loci can differ from those used to develop standard tools. For instance, in cohorts where hazel and green phenotypes are common, existing models often exhibit limited sensitivity, frequently yielding undefined results or misclassifying these shades [8]. This lack of resolution creates an investigative gap, as intermediate phenotypes represent a substantial portion of the population in both Southern Europe and North America. Despite the adoption of three‐category classification standards by international forensic proficiency testing organizations, the ability to reliably predict intermediate phenotypes in these specific demographic contexts remains a critical gap in the field.

This study investigates the possibility of improvement in FDP by implementing a machine learning‐based approach using the LightGBM framework [9]. We utilized an expanded panel of 93 pigmentation‐associated SNPs as the initial feature set, from which a final 37‐SNP predictive signature was derived, to capture the polygenic and pleiotropic nature of eye color while maintaining compatibility with modern sequencing workflows. This approach optimizes three‐category classification for blue, brown, and intermediate phenotypes. Our model was trained and validated on an Italian cohort and is publicly accessible via GenoEye, an interactive web application designed for forensic use. To address the *black‐box* challenge often associated with advanced algorithms, GenoEye also incorporates Shapley Additive explanations (SHAP) [10] values to provide interpretable predictions.

By focusing on the classification of intermediate eye colors, this work provides a practical approach to increasing diagnostic lift and enhancing phenotypic lead generation in Mediterranean and Southern European contexts. Furthermore, it offers a framework that can be extended to other admixed populations where intermediate shades are prevalent.

## 2 MATERIALS AND METHODS

### 2.1 Study cohort and genetic data

This study analyzed samples from a cohort of 376 Italian individuals whose recruitment and eye color phenotyping have been described previously [11]. Genotype data were generated using a custom‐designed SNP panel. All participants provided informed consent in accordance with the Declaration of Helsinki, and the research was approved by the relevant institutional ethics committee (approval code: HEC‐DSB/01–2023).

Iris color was phenotyped as described previously [11]. In brief, two independent observers classified eye color into three categories (blue, brown, and intermediate), with discrepancies resolved by a third observer through majority voting. Importantly, this phenotyping scheme was defined prior to the present study and was not modified during model development, ensuring that no optimization was performed with respect to phenotype definition. The intermediate category includes both green and hazel phenotypes, without further subdivision. This classification approach is consistent with commonly adopted frameworks in FDP, where eye color is assessed visually and intermediate phenotypes encompass non‐blue, non‐brown eye colors as well as heterogeneous iris patterns.

The initial selection of genetic markers for the SNP panel was based on an extensive literature review and forensic pigmentation panels. This resulted in a preliminary set of 111 variants previously associated with human pigmentation (Table S1). To genotype these markers, genomic DNA was extracted from available buccal swabs using the QIAamp DNA Mini Kit (Qiagen). Libraries were prepared using a custom Ion AmpliSeq panel and sequenced on an Ion S5 System. The resulting data were processed using Torrent Suite v5.2.2 software, and genotypes were called with Torrent Variant Caller v5.2.1.39.

A quality control (QC) protocol was subsequently applied to the newly generated data. Individuals with a sample call rate below 50% were excluded. Likewise, genetic variants with a call rate below 80% or a minor allele frequency (MAF) below 1% were removed.

After filtering, the final curated dataset included 93 informative SNPs across 363 individuals. The cohort was split into three non‐overlapping subsets, each corresponding to a distinct sample collection time point. These subsets were then used for model development and evaluation as follows: a training set of 249 individuals (68.6%), an internal validation set of 68 individuals (18.7%), and an external validation set of 46 individuals (12.7%).

### 2.2 Model development and hyperparameter optimization

A predictive model was developed using the LightGBM gradient boosting framework [9]. This tree‐based machine learning algorithm was selected because of its high efficiency and its ability to natively handle missing genotype data without requiring external imputation methods. Unlike many traditional models that require complete data sets, LightGBM learns the optimal branching direction for missing values during the training process. This feature is particularly relevant for forensic applications where DNA degradation often results in partial genetic profiles. By determining the best path for missing values based on the training data, the model can operate directly on incomplete data while preserving the original observations. This approach ensures that GenoEye remains functional even when specific markers in the 93 SNP panel cannot be successfully genotyped.

Model training and hyperparameter optimization were performed exclusively on the training dataset (*N* = 249). Hyperparameters were optimized using a 3‐fold cross‐validation scheme in combination with Bayesian optimization (BayesSearchCV), allowing systematic exploration of the parameter space while reducing the risk of overfitting (Table S2).

Importantly, the internal validation and external validation datasets were not used during hyperparameter tuning, ensuring a clear separation between model optimization and subsequent evaluation steps.

### 2.3 Final model construction and classification thresholding

Following hyperparameter optimization, a final LightGBM model was trained on the full training cohort (*N* = 249). During this stage, the internal validation dataset (*N* = 68) was used exclusively to implement early stopping, allowing independent monitoring of model convergence.

The same internal validation dataset was subsequently used to determine class‐specific probability thresholds for classification using Youden's *J* statistic (*J* = sensitivity + specificity − 1). For each class, the optimal cutoff was defined as the threshold maximizing the J statistic, resulting in three class‐specific thresholds: $J_{\text{blue}}$, $J_{\text{brown}}$ and $J_{\text{intermediate}}$.

The model outputs a normalized multiclass probability distribution for the three eye color categories $P\left(\text{blue}\right)$, $P\left(\text{brown}\right)$, and $P\left(\text{intermediate}\right)$, where the predicted probabilities sum to 1. Final classification was performed using a rule‐based framework based on these class‐specific thresholds, rather than independent binary classifiers. The classification logic is as follows:

- Blue: if $P\left(\text{blue}\right)>J_{\text{blue}}$ and $P\left(\text{brown}\right)\leq J_{\text{brown}}$
- Brown: if $P\left(\text{brown}\right)>J_{\text{brown}}$ and $P\left(\text{blue}\right)\leq J_{\text{blue}}$
- Intermediate: if $P\left(\text{intermediate}\right)>J_{\text{intermediate}}$, $P\left(\text{blue}\right)\leq J_{\text{blue}}$ and $P\left(\text{brown}\right)\leq J_{\text{brown}}$
- Undefined: otherwise.

This approach prioritizes the confident prediction of blue and brown phenotypes, classifying a sample as intermediate only in the absence of a strong signal for either blue or brown. Furthermore, the inclusion of an Undefined category ensures that samples with ambiguous genetic signals are not forced into a categorical prediction, thereby increasing the overall reliability of the results for forensic applications.

### 2.4 Model performance evaluation

The performance of the finalized model, complete with its optimized thresholds, was assessed on the unseen external validation dataset. This independent cohort was not used during any phase of model training, hyperparameter tuning, or threshold determination, ensuring an unbiased evaluation of the model's generalization capabilities.

Standard performance metrics were calculated, including sensitivity, specificity, and overall accuracy. The overall predictive power of the model for each class was quantified using the Area Under the Receiver Operating Characteristic curve (AUC). To ensure robust statistical evaluation, 95% confidence intervals (CIs) were generated using a bootstrapping procedure with 1000 iterations on the external validation dataset. This approach provides a reliable estimate of the model stability and performance across different potential sample distributions.

To assess model robustness to missing data, we simulated increasing levels of missingness by randomly masking SNPs at predefined proportions and evaluating predictive performance as a function of missing data. In addition, we assessed the impact of individual variants by iteratively removing each SNP from the model and measuring the resulting change in performance.

### 2.5 Model interpretability and feature importance

To elucidate the model's decision‐making process and move beyond a *black‐box* approach, we employed SHAP [10]. SHAP is a method based on game theory that enhances model interpretability by calculating feature importance values for each individual prediction, providing a unified and consistent measure of the contribution of each genetic marker.

The finalized LightGBM model was analyzed using the SHAP framework to identify the genetic markers with the greatest impact on its predictions. This analysis revealed a total of 37 features that were predictive for the accurate classification of blue, brown, and intermediate eye colors. The SHAP values allowed for a global understanding of the model behavior by ranking the features by their overall importance, effectively bridging the gap between complex machine learning algorithms and biological interpretability.

### 2.6 Interactive prediction application

To facilitate the use and exploration of our predictive model, an interactive web application was developed using the R Shiny framework [12]. This publicly accessible tool allows users to input sample genotype data and receive a real‐time eye color prediction based on the trained LightGBM model.

A key feature of the application is the integration of the SHAP framework to provide local, sample‐specific interpretability. For each prediction generated, the application visualizes the SHAP values, illustrating how each input feature contributed positively or negatively to the final classification decision. This allows users to not only receive a prediction but also to understand the specific genetic drivers behind that prediction for any given sample, a feature of particular relevance for forensic experts who must justify individual phenotypic leads.

## 3 RESULTS

### 3.1 Study cohort and genotype data

To construct a robust genetic panel for eye color prediction, an initial set of 111 variants was compiled based on an extensive review of pigmentation genetics (Table S1). The analysis was conducted on the Italian cohort previously described in [11]. After quality control filtering for both variants and samples, the final dataset included 363 individuals with high‐quality genotype information across 93 informative SNPs.

**FIGURE 1:**
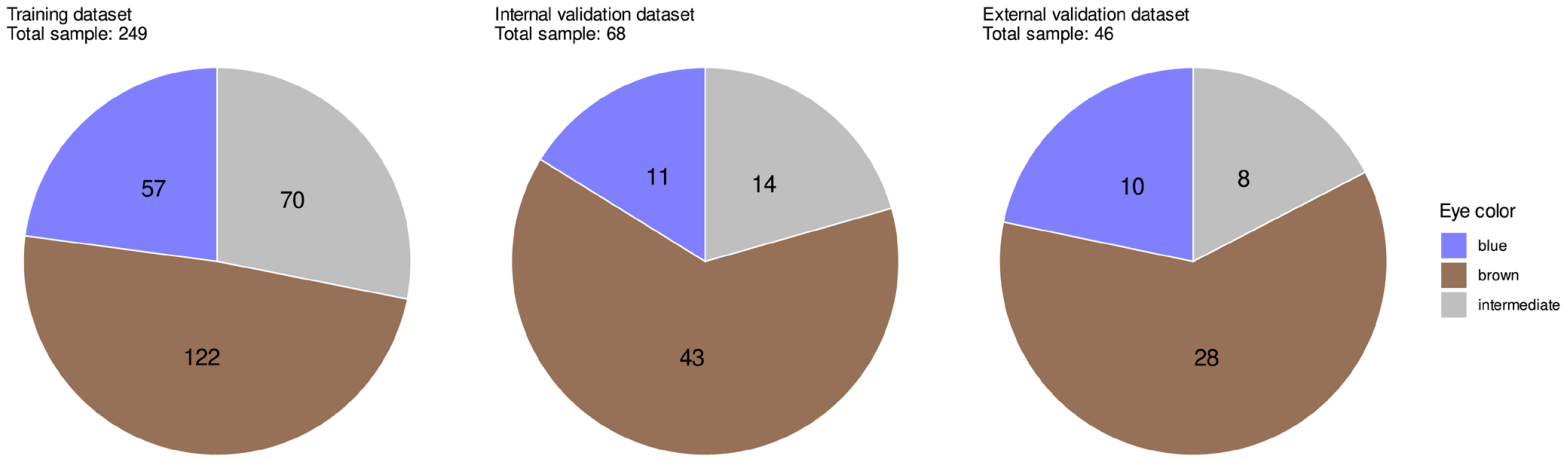
Distribution of eye color phenotypes across study cohorts. Pie charts illustrating the distribution of the three eye color phenotypes (blue, intermediate, and brown) across the training, internal validation, and external validation cohorts. Each chart displays the proportion of each class, with the total number of individuals and the count for each specific phenotype indicated.

The eye color distribution in the final cohort was 22% blue (*n* = 78), 53% brown (*n* = 193), and 25% intermediate (*n* = 92). For model development and evaluation, the dataset was partitioned into three independent subsets based on distinct collection time points: a training set (*n* = 249), an internal validation set (*n* = 68), and an external validation set (*n* = 46) (Figure 1). The phenotypic distribution remained consistent across subsets. Eye color categories were defined prior to this study using a standardized protocol and remained unchanged throughout all analyses, ensuring consistency in phenotype assignment.

### 3.2 Predictive model performance

**FIGURE 2:**
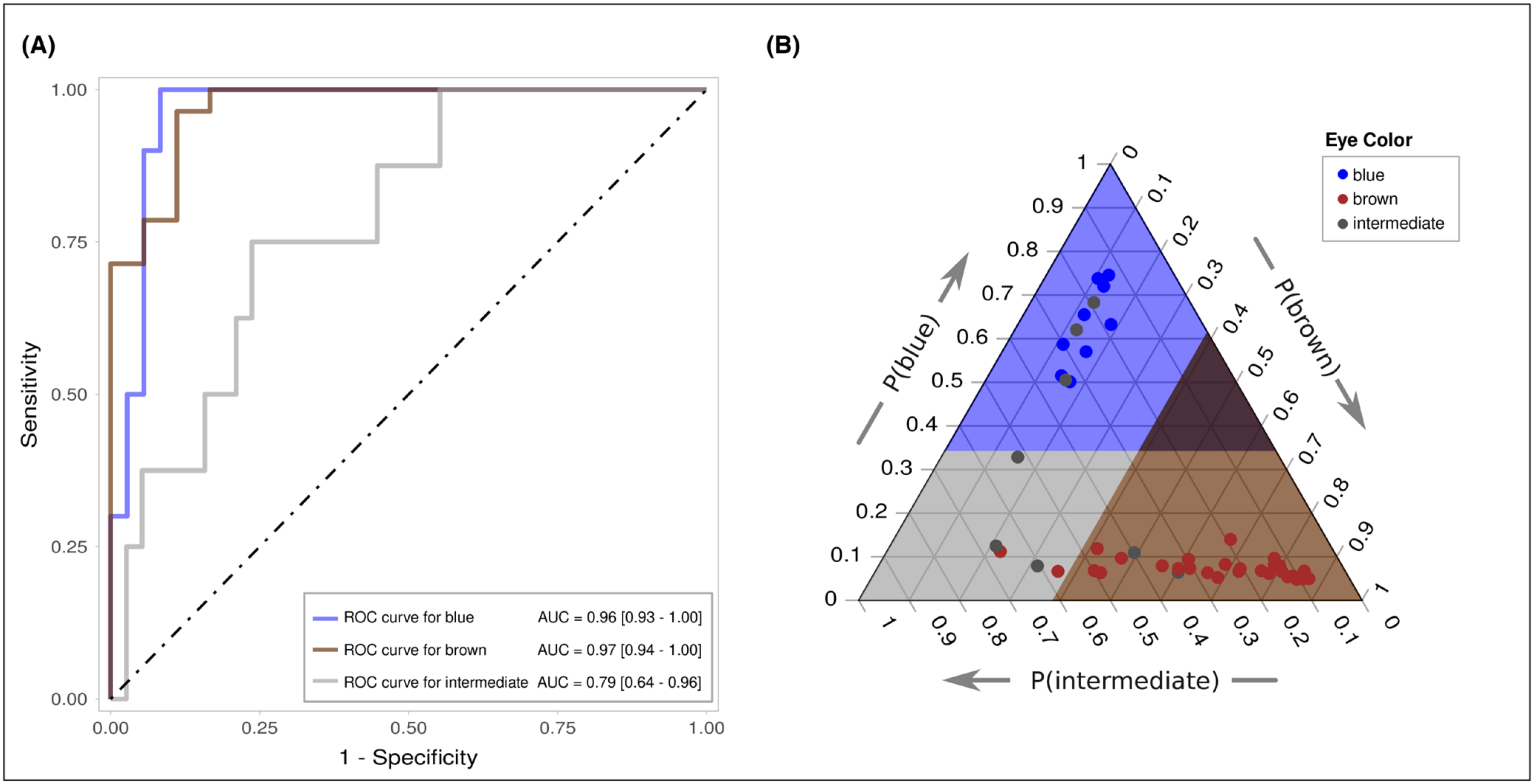
Predictive performance of the LightGBM model on the external validation cohort. (A) ROC curves for the prediction of blue, intermediate, and brown eye colors. AUROC values and their 95% confidence intervals, computed via a 1000‐iteration bootstrap procedure, are shown for each class. (B) Ternary plot visualizing the predicted probabilities for each individual in the external validation set. Each point represents an individual sample, and its position is determined by the three predicted probabilities generated by the model. The color of each point corresponds to the true, observed eye color phenotype. The background color delineates the decision regions for each predicted class: Blue, brown, intermediate (gray), and undefined (black), based on the optimized classification thresholds. Each point corresponds to a single individual (*N* = 46) in the external validation dataset.

A LightGBM model was developed using the training set, with hyperparameters tuned via 3‐fold cross‐validation and a Bayesian optimization approach. The classification thresholds were subsequently optimized using the internal validation set. The final model's performance was evaluated on the independent, external validation set. Following model building and feature‐importance assessment, the final GenoEye predictive signature comprised 37 SNPs derived from the 93 informative SNPs retained after quality control. Therefore, the performance metrics reported below refer to this finalized 37‐SNP GenoEye model. The model demonstrated strong predictive accuracy for the categorical classification of eye color, with high AUC values observed for all categories (Figure 2A). For the prediction of blue eyes, the model achieved an AUC of 0.96 (95% CI: 0.93–1.00), with a sensitivity of 1.00 and a specificity of 0.91. The prediction of brown eyes yielded an AUC of 0.97 (95% CI: 0.94–1.00), with 0.93 sensitivity and 0.87 specificity.

Notably, the model demonstrated a significant capacity to identify intermediate eye colors, achieving an AUC of 0.79 (95% CI: 0.64–0.96). Although the sensitivity for this challenging class was 0.38, the high specificity of 0.95 ensures a low false‐positive rate, which is a critical feature for reliable phenotypic lead generation in forensic contexts.

The overall accuracy of the model on the external validation set was 0.85. However, considering the moderately imbalanced distribution of eye color phenotypes in the cohort, standard accuracy can be an unreliable metric, as it can be inflated by high performance on the majority class while masking poor performance on minority classes. Therefore, to provide a more robust assessment, the balanced accuracy (computed as the average of sensitivity and specificity) was calculated for each class, yielding an overall mean balanced accuracy of 0.84. This consistency between standard and balanced accuracy supports the robustness of the model across all phenotypic categories. A comprehensive summary of all performance metrics, including the optimal cutoff thresholds, is provided in Table 1, while a detailed distribution of the predicted probabilities for all individuals in the external validation dataset is displayed in Figure 2B, where each point represents a single individual. No samples were assigned to the undefined category in the external validation dataset.

**TABLE 1:** Performance metrics of the GenoEye model on the external validation dataset.

Eye color	Youden's *J* statistic	AUROC	AUPRC	Balanced accuracy	Precision	Recall	Specificity
Blue	0.34	0.96 [0.93–1.00]	0.84 [0.69–1.00]	0.95 [0.93–0.97]	0.77 [0.67–0.83]	1.00 [1.00–1.00]	0.91 [0.88–0.93]
Brown	0.38	0.97 [0.94–1.00]	0.98 [0.96–1.00]	0.90 [0.87–0.94]	0.93 [0.90–0.96]	0.93 [0.90–1.00]	0.87 [0.82–0.92]
Intermediate	0.19	0.79 [0.64–0.96]	0.38 [0.05–0.63]	0.66 [0.61–0.70]	0.60 [0.51–1.00]	0.38 [0.29–0.43]	0.95 [0.93–1.00]

*Note*: For each eye color category, the optimal classification threshold derived from Youden's J statistic is reported together with the corresponding performance metrics obtained on the external validation set. AUROC (area under the receiver operating characteristic curve) and AUPRC (area under the precision–recall curve) are presented with 95% confidence intervals, estimated using a bootstrap procedure with 1000 iterations. Balanced accuracy, precision, recall (sensitivity), and specificity are computed based on the final rule‐based classification framework using class‐specific thresholds.

### 3.3 Robustness to missing data and key variant removal

**FIGURE 3:**
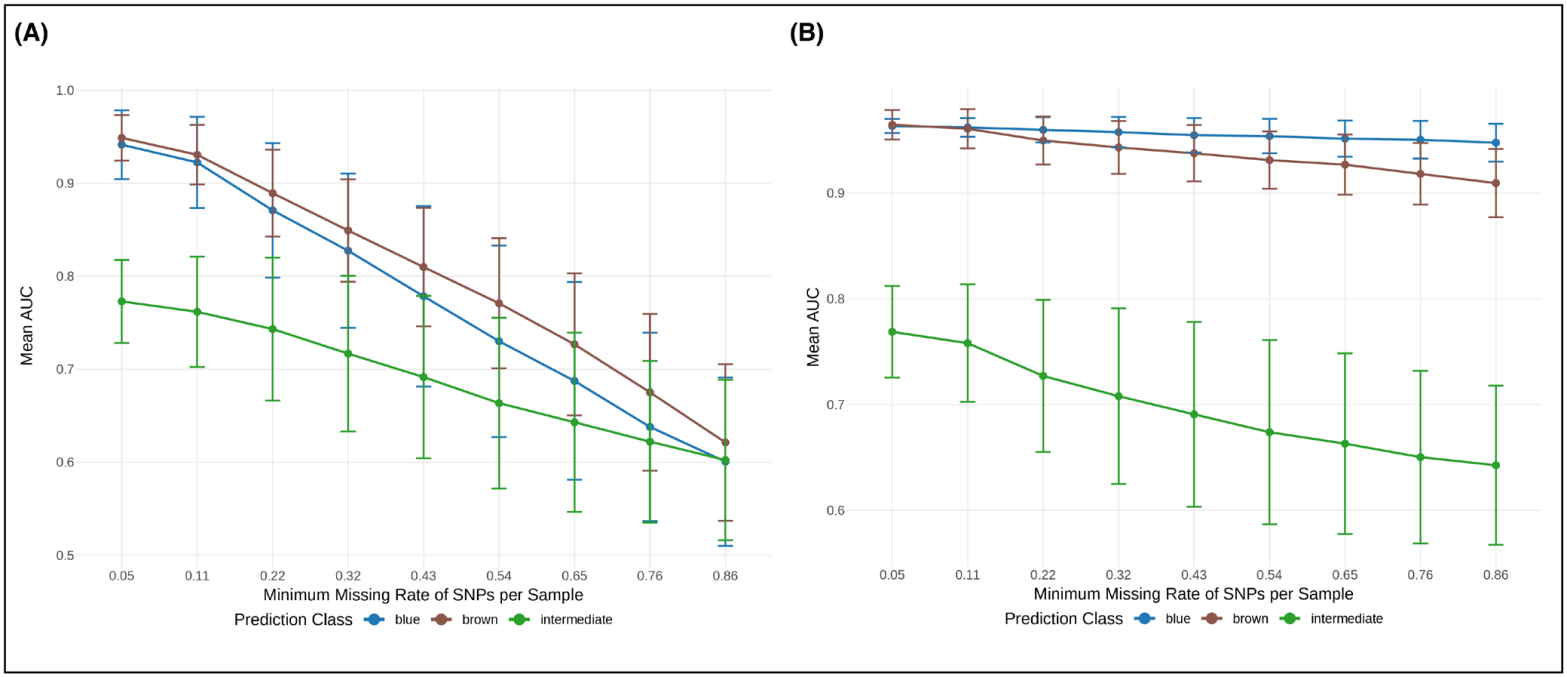
Impact of missing data on GenoEye predictive performance. Mean AUC values for the prediction of blue, brown, and intermediate eye color are shown as a function of the minimum missing rate of SNPs per sample. Increasing missingness levels were simulated by progressively removing SNP information, and model performance was evaluated across these scenarios. (A) Missingness applied across all SNPs in the panel. (B) Missingness applied while retaining the key variant rs1129038. Error bars represent variability across simulations.

To evaluate the robustness of the model to incomplete genetic profiles, we simulated increasing levels of missing data by progressively masking SNPs and assessing predictive performance as a function of the proportion of missing variants (Figure 3A). The results show that model performance remains stable up to moderate levels of missingness, with only a gradual decrease observed at higher levels.

To further investigate the contribution of individual variants, we evaluated the effect of removing each SNP from the model and measuring the resulting change in predictive performance (Table S3). The model showed limited sensitivity to the removal of most variants, with only minor decreases in AUC observed. Notably, rs1129038 was identified as the variant whose removal resulted in the largest reduction in performance in the trained GenoEye model, particularly for blue–brown discrimination. Because rs1129038 is in strong linkage disequilibrium with the functionally characterized HERC2 variant rs12913832 [13], this result is best interpreted as reflecting the importance of the broader HERC2/OCA2 regulatory signal rather than a distinct functional role of rs1129038 itself.

To specifically assess the contribution of this key HERC2/OCA2 locus under incomplete profiles, we repeated the missingness analysis while keeping rs1129038 always observed (Figure 3B). Compared with the fully random masking scenario, predictive performance was better preserved across missingness levels, indicating that part of the performance loss observed under random missingness is driven by the occasional absence of rs1129038. This effect was more pronounced for the discrimination of blue and brown eye color, while the performance for intermediate phenotypes showed limited variation across conditions. Since rs1129038 is in strong linkage disequilibrium with the functionally characterized rs12913832 variant, this result is interpreted as reflecting the importance of the broader HERC2/OCA2 regulatory signal in the trained model.

Together, these results indicate that the model is generally robust to incomplete genotype data and to the absence of most individual SNPs. While highly informative variants such as rs1129038 play a key role in discriminating extreme phenotypes, the prediction of intermediate eye color appears to rely on a broader combination of markers.

### 3.4 Comparison with the HIrisPlex‐S system

To provide a direct benchmark, we evaluated the widely used HIrisPlex‐S system (https://hirisplex.erasmusmc.nl/) on our external validation cohort (*N* = 46). We first compared the intrinsic discriminative ability of the two models using threshold‐independent metrics.

**FIGURE 4:**
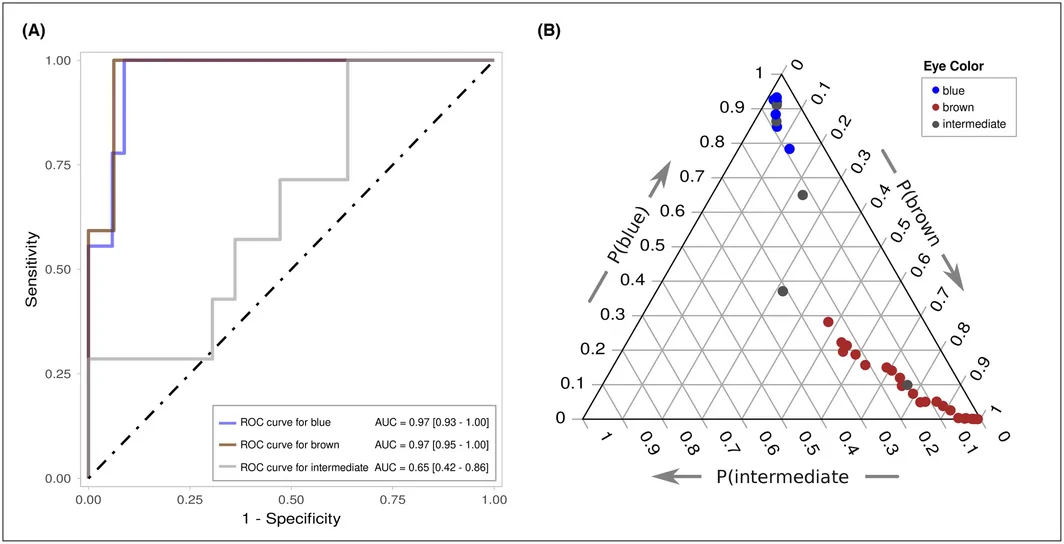
Performance of the HIrisPlex‐S system on the external validation cohort for comparison. (A) ROC curves for predictions made by the HIrisPlex‐S system on the external validation set. AUC values and their 95% confidence intervals were computed using a 1000‐iteration bootstrap procedure. (B) Ternary plot showing the predicted probabilities generated by the HIrisPlex‐S webtool for each sample in the external validation set. Each point's position reflects the predicted probability for blue, intermediate, and brown classes, while its color indicates the true phenotype.

HIrisPlex‐S demonstrated high performance for the prediction of extreme phenotypes (blue and brown, both yielding an AUC of 0.97). However, its predictive power for the intermediate class was notably lower, with an AUC of 0.65 (95% CI: 0.42–0.86), compared to the 0.79 (95% CI: 0.64–0.96) achieved by GenoEye (Figure 4).

To assess how model outputs translate into categorical predictions, we evaluated multiple decision strategies applied consistently to both models (Table 2 and Figure S1).

**TABLE 2:** Performance metrics comparison of eye color prediction models on the external validation dataset.

Eye color	Youden's *J* statistic	Balanced accuracy	Precision	Recall	Specificity
HIrisPlex‐S–GenoEye threshold method					
Blue	0.11	0.75	0.36	1.00	0.50
Brown	0.81	0.78	1.00	0.55	1.00
Intermediate	0.13	0.53	0.33	0.14	0.92
GenoEye–0.50 threshold					
Blue	–	0.95	0.77	1.00	0.90
Brown	–	0.86	0.96	0.77	0.93
Intermediate	–	0.67	0.40	0.50	0.84
HIrisPlex‐S–0.50 threshold					
Blue	–	0.94	0.69	1.00	0.88
Brown	–	0.94	0.96	0.96	0.92
Intermediate	–	0.63	0.67	0.28	0.26
GenoEye–max threshold					
Blue	–	0.95	0.77	1.00	0.87
Brown	–	0.84	0.92	0.82	0.87
Intermediate	–	0.62	0.38	0.38	0.87
HIrisPlex‐S–max threshold					
Blue	–	0.91	0.60	1.00	0.82
Brown	–	0.95	0.96	1.00	0.90
Intermediate	–	0.50	NA	0	1.00

*Note*: Comparative evaluation of GenoEye and HIrisPlex‐S using the external validation set. To assess performance under different classification criteria, metrics were computed using three distinct probability thresholding approaches: (i) GenoEye threshold, where the specific thresholding logic, derived by maximizing Youden's J statistic on the internal validation dataset, is applied to the HIrisPlex‐S probabilities for standardized comparison; (ii) 0.5 threshold, where ‘blue’ and ‘brown’ are predicted if their respective probabilities exceed 0.5, with ‘intermediate’ assigned otherwise; (iii) max threshold, where the predicted eye color is determined strictly by the category with the highest probability. Classification metrics, including balanced accuracy, precision, recall (sensitivity), and specificity, are reported for each model across these thresholding rules.

First, categorical predictions were assigned based on the highest predicted probability. Under this common decision framework, HIrisPlex‐S failed to correctly classify any of the samples belonging to the intermediate category (recall = 0), whereas GenoEye retained measurable sensitivity for this class (recall = 0.38), while maintaining high performance for blue eye color. Under this rule, however, the gain in intermediate recall was accompanied by lower brown‐eye recall for GenoEye than for HIrisPlex‐S.

We next explored a simple threshold‐based rule in which blue and brown predictions were assigned only when their predicted probability exceeded 0.5, and all remaining samples were classified as intermediate. Under this setting, both models showed an increase in intermediate assignments. However, GenoEye maintained higher recall (0.50 vs. 0.28) and balanced accuracy (0.67 vs. 0.63) for the intermediate class compared to HIrisPlex‐S, although brown‐eye recall was lower under this rule.

Finally, we applied a threshold‐based classification approach using class‐specific cutoffs derived from Youden's J statistic to both models. While this strategy corresponds to the primary classification framework used in GenoEye, applying the same approach to HIrisPlex‐S resulted in a decrease in performance, particularly for intermediate eye color (balanced accuracy = 0.53, recall = 0.14), as well as reduced stability for blue and brown predictions. Notably, the combination of GenoEye with *J*‐statistic‐based thresholding yielded the highest overall classification performance among all evaluated configurations, providing the best balance between improved intermediate sensitivity and preserved performance for blue and brown eye color.

As an additional threshold‐independent control analysis, we trained a constrained LightGBM model using only the six eye‐color SNPs included in IrisPlex. This six‐SNP model showed AUC values comparable to HIrisPlex‐S across the three eye‐color classes, including intermediate eye color, indicating that LightGBM alone does not improve threshold‐independent discrimination when restricted to the IrisPlex marker set (Figure S2).

These observations are consistent with previously reported performance metrics for HIrisPlex‐S derived from a large dataset of 9466 individuals, where intermediate eye color prediction shows extremely low sensitivity (0.001 ± 0.003), reflecting a strong polarization of the model towards extreme phenotypes. Despite the difference in sample size, the lack of overlap between the sensitivity confidence interval of GenoEye [0.29–0.43] and that reported for HIrisPlex‐S [0.000–0.004] suggests a significant difference in categorical accuracy for non‐extreme phenotypes (*p* = 0.0023, Fisher's Exact Test).

Under the *J*‐statistic‐based classification framework, the observed differences in sensitivity have a direct impact on the Positive Predictive Value (PPV), particularly for intermediate phenotypes. In a Mediterranean population context, assuming a prevalence of 20% for intermediate eye color, GenoEye achieved a PPV of 65.5%. Even at a lower prevalence (5%), the model maintained a PPV of 28.6%, supporting its potential utility in forensic applications. This improvement is directly linked to the higher sensitivity achieved by GenoEye for intermediate phenotypes, whereas the consistently lower sensitivity observed for HIrisPlex‐S limits its practical ability to identify this class.

Overall, these results suggest that, while HIrisPlex‐S is optimized for high overall accuracy in polarized populations, GenoEye more effectively captures the biological signal of intermediate phenotypes across different decision strategies, providing diagnostic lift in the evaluated Italian cohort, which reflects a Southern European context where intermediate eye‐color phenotypes are relatively common.

### 3.5 Model interpretability and key predictive features

**FIGURE 5:**
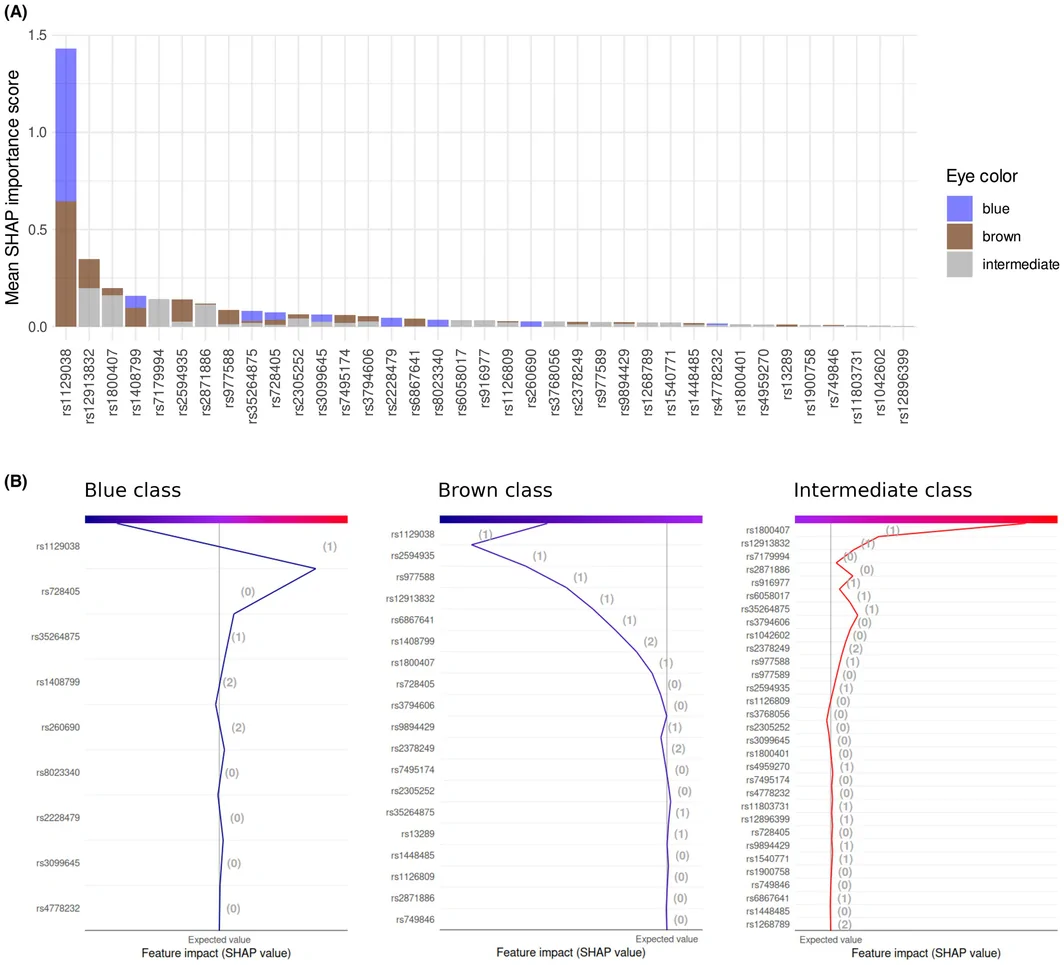
Feature importance analysis using SHAP. (A) Bar plot illustrating the mean absolute SHAP values for the 37 most predictive genetic variants in the LightGBM model. The bars are stratified by eye color class, showing the specific contribution of each variant to the prediction of blue, intermediate, and brown phenotypes. This visualization highlights the key genetic drivers for each classification task and allows for a global interpretation of the model's behavior. (B) SHAP decision plot for an example individual (Sample4, also represented in Figure 6), showing how the values of specific features push the model's prediction from the base value to its final output for this single case.

To understand the model's decision‐making process, we employed the SHAP framework [10]. This analysis identified a total of 37 SNPs as being the most influential for predicting eye color. The global SHAP summary plot confirmed the central role of the HERC2/OCA2 region in GenoEye predictions. Within this locus, rs1129038 showed the strongest contribution to blue‐brown discrimination in the trained model, whereas the functionally characterized variant rs12913832 was retained and contributed more prominently to intermediate eye‐color prediction (Figure 5). Additional pigmentation markers, including rs1800407 (OCA2), also contributed to the prediction of the intermediate phenotype.

This pattern is consistent with the well‐established role of the HERC2/OCA2 region in eye color determination, where rs12913832 is known to be the major genetic determinant of the blue versus brown eye color axis. The prominence of rs1129038 in GenoEye should therefore not be interpreted as evidence that this variant is functionally primary. Rather, it likely reflects its strong linkage disequilibrium with rs12913832, allowing the model to capture the same underlying regulatory signal. Notably, both variants were retained in the model, but their contributions differed across classes. In particular, rs1129038 contributed more strongly to the discrimination of blue and brown eye color, whereas rs12913832 showed a greater contribution to the prediction of intermediate phenotypes. This behavior is consistent with the ability of tree‐based models to exploit correlated variants differently depending on the classification task.

This analysis not only confirmed the high importance of well‐established pigmentation markers but also highlighted which specific variants most effectively aid in the discrimination between the three eye color classes. In particular, the class‐specific contribution of rs1129038 and rs12913832 illustrates how correlated markers within the HERC2/OCA2 locus may support different aspects of eye‐color prediction in the model. In addition, the contribution of multiple variants supports the polygenic nature of intermediate eye color, which likely arises from the combined effect of several loci with more subtle regulatory roles.

### 3.6 Interactive prediction and interpretation via web application

**FIGURE 6:**
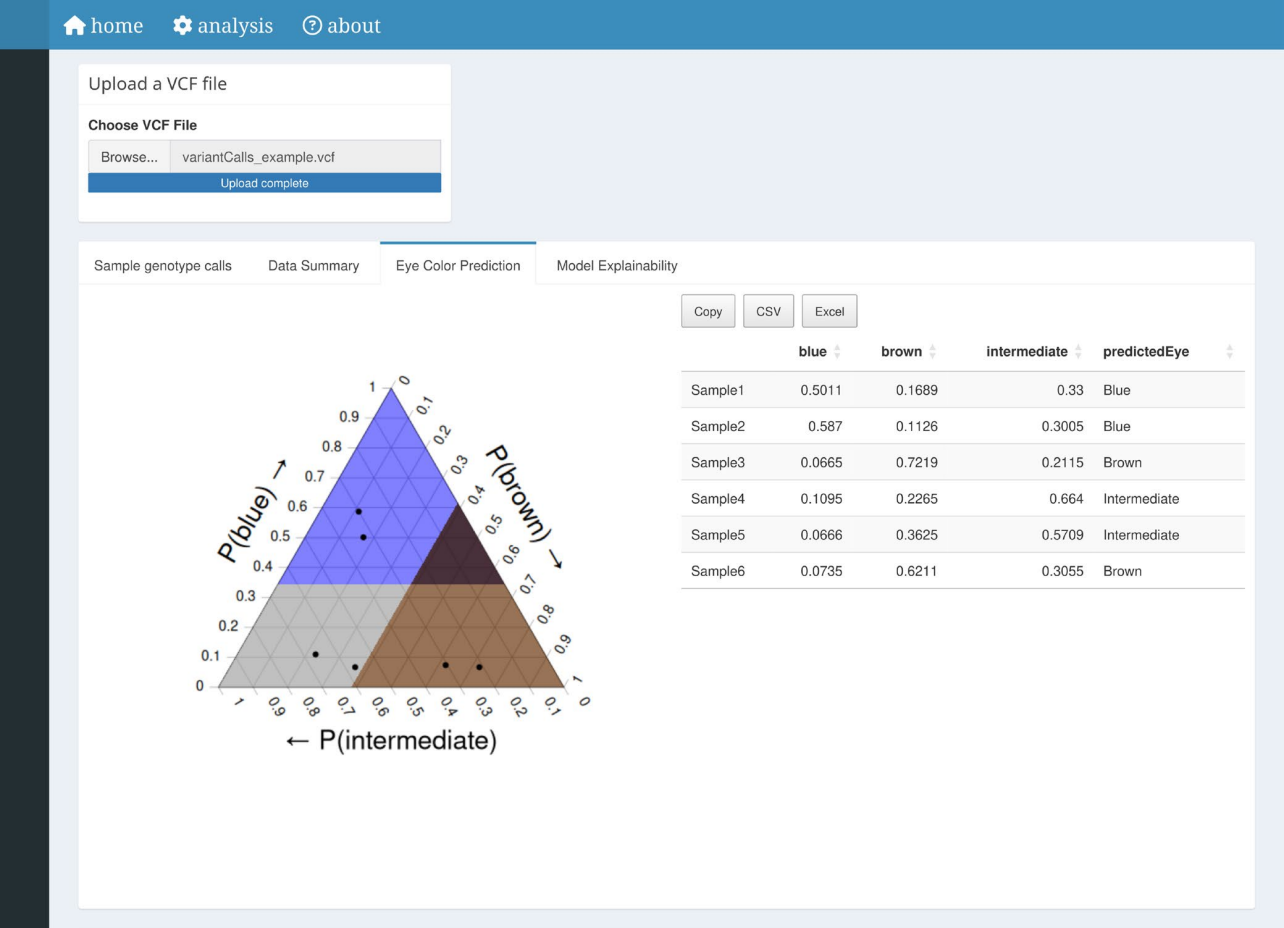
The GenoEye interactive web application. The screenshot shows the ‘Analysis’ page where users upload a VCF file containing variant genotypes (top). The main panel displays the ‘Eye Color Prediction’ tab for an example analysis of six samples. On the right, a table lists the predicted probabilities for each class (blue, brown, and intermediate) and the final eye color prediction for each sample. These predictions are also visualized in a ternary plot on the left.

To provide a practical and accessible tool for researchers, we developed and deployed an interactive web application, GenoEye, built with the R Shiny framework (Figure 6). The application allows users to perform real‐time eye color prediction by inputting genotype data for the relevant markers. In addition to providing a classification, the tool integrates SHAP to deliver instance‐specific explanations. For each sample, the application generates a SHAP decision plot, which visualizes how each feature quantitatively contributes to pushing the model's prediction towards a specific eye color (Figure 5B). This feature makes the model's reasoning transparent, allowing users to understand the genetic drivers behind any individual prediction and facilitating the expert interpretation required in forensic reporting.

## 4 DISCUSSION

In this study, we developed and evaluated GenoEye, a machine learning framework designed for eye color prediction with a specific focus on the Italian population. A primary objective of this work was to investigate potential improvements in classifying *intermediate* phenotypes, which consistently represent a challenge for current FDP tools. Our results indicate that the model achieved an overall accuracy of 0.84 and an AUC of 0.79 for the intermediate category within our independent validation cohort. While these findings are based on a limited sample size, they indicate a measurable improvement in the identification of intermediate phenotypes compared with current standard forensic tools. This is particularly relevant for other regions with significant phenotypic diversity, such as North America, where intermediate shades like hazel and green represent a substantial portion of the population and pose similar challenges for forensic identification.

These results appear consistent with recent findings, such as those reported in [8], which suggested that machine learning models combined with optimized SNP panels can improve the prediction of non‐blue or non‐brown eye color phenotypes. Similar conclusions have been reported in a recent independent study by Martínez et al. [14], who applied multiple machine learning approaches to eye color prediction in a Latin American cohort. Despite differences in population background, marker selection, and modeling strategies, their results support the potential of machine learning‐based frameworks to better capture intermediate eye color variation. However, there is a distinction in methodological design and final objective between these studies and our work. While previous research primarily focused on the comparative evaluation of different machine learning models and their predictive potential, without defining a single standardized or directly deployable prediction framework for forensic application, our study was designed to produce a practical and interpretable model for direct forensic use. By utilizing three distinct and independent cohorts, we developed a single finalized model whose performance was assessed on entirely unseen data. Consequently, the metrics we report reflect the realistic performance of this specific predictive tool in an operational forensic context.

A comparison with the HIrisPlex‐S system [4] within our validation set showed that GenoEye achieves comparable performance for blue and brown eye color while demonstrating improved ability to identify intermediate phenotypes. In particular, GenoEye reached an AUC of 0.79 for the intermediate category, compared to 0.65 observed for the benchmark model. To ensure a fair comparison, we distinguished between threshold‐independent performance, assessed using AUC, and categorical predictions obtained under different decision strategies. When applying consistent classification frameworks to both models, including highest‐probability assignment, fixed thresholds, and class‐specific thresholds derived from Youden's *J* statistic, GenoEye consistently retained improved sensitivity for intermediate phenotypes while blue and brown performance remained high overall, particularly under the final optimized classification framework.

These results indicate that the observed improvement is not solely driven by the choice of decision thresholds, but reflects differences in the underlying probabilistic outputs of the models. In particular, the probability distributions generated by GenoEye appear to better capture the signal associated with non‐extreme phenotypes.

While thresholds derived from Youden's *J* statistic are inherently dataset‐dependent and may require re‐calibration in other populations, in this study they were estimated on an independent internal validation set and evaluated on a separate external dataset, thereby reducing the risk of overfitting.

An additional aspect to consider is the definition of intermediate eye color, which is inherently heterogeneous and may include green, hazel, and mixed pigmentation patterns. Differences in classification criteria across studies may therefore influence reported performance. In this study, eye color categories were defined using a previously established protocol [11] and were not modified during the analysis, ensuring that model performance is not driven by post hoc adjustments of phenotype definitions but instead reflects the model's ability to capture the biological variation underlying non‐extreme eye color phenotypes.

The performance of GenoEye likely derives from the synergy between an expanded feature space and the LightGBM algorithm. The final GenoEye predictive signature includes 37 SNPs selected from the initial set of 93 informative variants retained after quality control, a selection that aims to capture the polygenic architecture of pigmentation while balancing predictive power with forensic practicalities. This 37‐SNP signature represents an approximately six‐fold increase in marker number relative to the six eye‐color SNPs used by IrisPlex. Consistent with this interpretation, a constrained LightGBM model trained using only the six IrisPlex SNPs produced AUC values comparable to HIrisPlex‐S across the three eye‐color classes, indicating that the classifier alone does not account for the improved performance of the full GenoEye model. By including markers that may exhibit pleiotropic effects across different pigmentation traits, such as skin and hair color, the expanded marker set potentially accounts for subtle genetic signals and shared biological pathways that influence iris pigmentation [15]. Notably, this final 37‐SNP signature highlights that the model leverages both well‐established pigmentation loci and additional variants contributing to finer phenotypic variation, particularly for intermediate eye color. While extensive genome‐wide association studies, such as the work by Simcoe and colleagues [7], have documented over 100 variants associated with eye color, the evaluation of these additional markers represents a possible direction for future research. Assessing broader genetic datasets may further enhance the sensitivity of machine learning‐based models for intermediate phenotypes while preserving the conservative decision criteria required in forensic applications.

Beyond predictive accuracy, a fundamental aspect of our work is its transparency. To address the concerns regarding opaque predictive methods often associated with machine learning, we integrated the SHAP framework. This approach moves beyond the traditional black box limitation by providing a clear biological context for each prediction through the GenoEye web application. In a forensic context, the ability to justify a phenotypic lead through local interpretability is essential for the acceptance of advanced algorithms by practitioners and legal experts.

We recognize several limitations that warrant a cautious interpretation of these results. An important practical aspect of FDP is the presence of missing data, which frequently occurs in degraded samples. In this study, the model showed stable performance up to moderate levels of missingness, with a gradual decrease at higher levels. Consistent with this, the analysis of individual SNP removal indicated that the model is generally robust to the absence of single variants, with the exception of rs1129038, whose removal results in a marked decrease in performance, particularly for blue‐brown discrimination. Given the strong linkage disequilibrium between rs1129038 and the functionally characterized HERC2 variant rs12913832, this effect is best interpreted as reflecting the importance of the broader HERC2/OCA2 regulatory signal in the trained model, rather than a distinct functional role of rs1129038 itself. These results indicate that the model can operate with incomplete genetic profiles, although predictions should be interpreted with reduced confidence when highly informative variants within the HERC2/OCA2 region are missing. To support practical use, the GenoEye web tool provides warnings based on missing data patterns. Of note, although the model natively handles missing data, a formal comparison with imputation‐based approaches was beyond the scope of this study and would be required to assess relative performance.

In addition, the external validation set included a small number of intermediate samples, and these performance metrics should be considered preliminary. Nevertheless, the concordance between our findings and recent machine learning‐based studies conducted in other admixed populations supports the general applicability of machine learning‐driven approaches for improving intermediate eye color prediction. Furthermore, while the focus on an Italian cohort addresses a specific gap for Southern European populations, the generalizability of the model to more diverse global populations remains to be established. However, the biological similarities and shared genetic architecture of pigmentation suggest that our approach could be highly applicable to other demographic contexts, including the admixed populations of North America, where the prevalence of complex intermediate eye shades is comparable to that of Mediterranean groups.

In conclusion, GenoEye offers an interpretable and accessible framework for eye color prediction that demonstrates a consistent and reproducible improvement in the classification of intermediate phenotypes, a long‐standing challenge in FDP. While further validation on larger cohorts is essential, this study provides a foundation for the use of advanced machine learning to enhance forensic phenotypic leads.

## CONFLICT OF INTEREST STATEMENT

The authors declare no conflicts of interest.

## Supporting information


**Figure S1.** Comparison of categorical decision regions for GenoEye and HIrisPlex‐S. Ternary plots showing predicted probabilities for blue, brown, and intermediate eye color in the external validation set under different categorical assignment strategies. The upper panels show GenoEye predictions using the 0.50 threshold and max‐probability assignment rules. The lower panels show HIrisPlex‐S predictions using the 0.50 threshold, max‐probability assignment, and the GenoEye threshold method. Each point represents one individual, and colors indicate the observed eye color phenotype. Shaded regions indicate the predicted decision areas for blue, brown, and intermediate eye color under each classification rule.
**Figure S2.** Performance of the constrained six‐SNP LightGBM model. ROC curves and AUC values for a LightGBM model trained using only the six eye‐color SNPs included in IrisPlex and evaluated on the external validation set.

**Table S1.** All candidate SNP markers selected from literature resources. The table indicates each SNP's inclusion in other established panels.
**Table S2.** Model's hyperparameters.
**Table S3.** Performance of GenoEye model excluding single SNPs.

## Data Availability

The application has been implemented as a freely available web‐based tool and is accessible at: https://bcglab.cibio.unitn.it/genoEye.
