# Supplementary material for: GenoEye: A machine learning‐based framework for the prediction of intermediate eye color phenotypes
Source: J Forensic Sci. 2026 Jul 2;71(5):2145–56. doi: 10.1111/1556-4029.70403 (PMC13534952; doi:10.1111/1556-4029.70403)
Supplement: Supplementary file 1 [file JFO-71-2145-s002.docx]

FIGURE S1	Comparison of categorical decision regions for GenoEye and HIrisPlex-S.

Ternary plots showing predicted probabilities for blue, brown, and intermediate eye color in the external validation set under different categorical assignment strategies. The upper panels show GenoEye predictions using the 0.50 threshold and max-probability assignment rules. The lower panels show HIrisPlex-S predictions using the 0.50 threshold, max-probability assignment, and the GenoEye threshold method. Each point represents one individual, and colors indicate the observed eye color phenotype. Shaded regions indicate the predicted decision areas for blue, brown, and intermediate eye color under each classification rule.

FIGURE S2	Performance of the constrained six-SNP LightGBM model.

ROC curves and AUC values for a LightGBM model trained using only the six eye-color SNPs included in IrisPlex and evaluated on the external validation set.
